# Preparation of Low-Diacylglycerol Cocoa Butter Equivalents by Hexane Fractionation of Palm Stearin and Shea Butter

**DOI:** 10.3390/molecules26113231

**Published:** 2021-05-27

**Authors:** Jihyun Hwang, Heeju Jun, Seoye Roh, Seong Jae Lee, Jeong Min Mun, Seung Wook Kim, Min-Yu Chung, In-Hwan Kim, Byung Hee Kim

**Affiliations:** 1 Department of Food and Nutrition, Sookmyung Women’s University, Seoul 04310, Korea; hyun8736@gmail.com (J.H.); gmlwn0527@naver.com (H.J.); shtjdp1@naver.com (S.R.); 2 Research Center, Ottogi Corporation, Gyeonggi 14060, Korea; sjlee0218@ottogi.co.kr (S.J.L.); jmmun0174@ottogi.co.kr (J.M.M.); kimsw@ottogi.co.kr (S.W.K.); 3 Korea Food Research Institute, Jeonbuk 55365, Korea; mic07002@kfri.re.kr; 4 Department of Food and Nutrition, Korea University, Seoul 02841, Korea; k610in@korea.ac.kr

**Keywords:** cocoa butter equivalents, diacylglycerols, hexane fractionation, palm stearin, shea butter

## Abstract

Herein, we prepared 1,3-dipalmitoyl-2-oleoyl glycerol (POP)-rich fats with reduced levels of diacylglycerols (DAGs), adversely affecting the tempering of chocolate, via two-step hexane fractionation of palm stearin. DAG content in the as-prepared fats was lower than that in POP-rich fats obtained by previously reported conventional two-step acetone fractionation. Cocoa butter equivalents (CBEs) were fabricated by blending the as-prepared fats with 1,3-distearoyl-2-oleoyl glycerol (SOS)-rich fats obtained by hexane fractionation of degummed shea butter. POP-rich fats achieved under the best conditions for the fractionation of palm stearin had a significantly lower DAG content (1.6 *w/w*%) than that in the counterpart (4.6 *w/w*%) prepared by the previously reported method. The CBEs fabricated by blending the POP- and SOS-rich fats in a weight ratio of 40:60 contained 63.7 *w/w*% total symmetric monounsaturated triacylglycerols, including 22.0 *w/w*% POP, 8.6 *w/w*% palmitoyl-2-oleoyl-3-stearoyl-*rac*-glycerol, 33.1 *w/w*% SOS, and 1.3 *w/w*% DAGs, which was not substantially different from the DAG content in cocoa butter (1.1 *w/w*%). Based on the solid-fat content results, it was concluded that, when these CBEs were used for chocolate manufacture, they blended with cocoa butter at levels up to 40 *w/w*%, without distinctively altering the hardness and melting behavior of cocoa butter.

## 1. Introduction

Cocoa butter, the main ingredient of chocolate, is an edible fat predominantly composed of symmetric monounsaturated triacylglycerols (SMUT), such as 1,3-dipalmitoyl-2-oleoyl-glycerol (POP), palmitoyl-2-oleoyl-3-stearoyl-*rac*-glycerol (POS), and 1,3-distearoyl-2-oleoyl-glycerol (SOS) [1]. POS (36–41 *w/w*%) is the most abundant SMUT in cocoa butter, followed by SOS (25–31 *w/w*%) and POP (15–19 *w/w*%) [1]. Due to the high cost and fluctuations in the supply and demand of natural cocoa butter, cocoa butter equivalents (CBE) are used as an alternative to cocoa butter in the manufacture of chocolate products because they are prepared from low-cost sustainable vegetable fats and oils including palm oil [2]. CBEs are edible fats with a triacylglycerol (TAG) composition similar to that of natural cocoa butter; therefore, CBEs blend with cocoa butter without altering its physical properties [2]. Commercial CBEs are commonly produced by blending POP-rich fats, which are prepared by fractionation of palm olein, with SOS-rich fats prepared by fractionation of shea butter or *sn*-1,3-specific lipase-catalyzed interesterification of high-oleic sunflower oil with stearic acid ethyl esters [3].

During chocolate manufacture, tempering of cocoa butter is a pre-crystallization process that involves mixing and cooling the chocolate under controlled conditions to obtain cocoa butter crystallized in a preferred polymorphic form, i.e., form V (also called form β_2_); this process endows the final product with desirable sensory attributes, such as a glossy appearance, appropriate snap, and satisfying melt-in-the-mouth texture [4]. Contents of the chemical constituents (e.g., acylglycerols, emulsifiers, and sugars) present in chocolate affect the rate of transition of cocoa butter from a less stable polymorphic form to a more stable form, which is one of the crucial factors determining the tempering attributes of chocolate [4]. Previously, several studies have reported that the presence of diacylglycerols (DAG) in cocoa butter or CBEs delays the transition of high-melting TAGs, including SMUTs, into more stable crystalline structures, causing difficulties in the tempering of the cocoa butter blended with CBEs [5,6]. The effects of monoacylglycerols—which are another type of partial acylglycerols, along with DAGs, in the tempering of cocoa butter—have not been reported previously. Thus, preparation of CBEs with reduced levels of DAGs is required for chocolate manufacture because the occurrence of DAGs in natural cocoa butter is unavoidable.

In our previous studies [7,8], we demonstrated that POP-rich fats for CBE formulation can be prepared by two-step acetone fractionation of palm stearin, which is a potential cost-effective material. However, we recently reproduced POP-rich fats from palm stearin under the same fractionation conditions as previously reported [7] and found that the DAG content in these fats ranged from 4 to 5 *w/w*%, which was higher than that in palm stearin (~3 *w/w*%). The elevated level of DAGs was attributed to the concentration of DAGs in the liquid phase during the first step of the two-step acetone fractionation.

When *n*-hexane, with polarity lower than that of acetone, was used as a solvent for fractionation, it concentrated more polar lipids than TAGs, such as DAG, monoacylglycerols, and fatty acids (FAs), in the solid phase rather than in the liquid phase [9,10]. This suggests that *n*-hexane can be an appropriate solvent for the two-step fractionation of palm stearin to fabricate POP-rich fats and the resultant CBEs containing reduced levels of DAGs compared to those in the products reported in our previous studies [7,8]. SOS-rich fats have been obtained from shea butter or high-oleic high-stearic sunflower oil by hexane fractionation [11,12]. However, to the best of our knowledge, no studies have reported the application of hexane fractionation techniques for the preparation of POP-rich fats from fats and oils including palm stearin. Moreover, *n*-hexane is the most widely used organic solvent in the edible oil extraction industry. Nevertheless, it has not been commonly used as the solvent for the fractionation of edible fats and oils. In South Korea, unlike acetone, the use of *n*-hexane is not legally permitted in the fractionation process. The aim of this study was to prepare POP-rich fats with reduced levels of DAGs by two-step hexane fractionation of palm stearin and to produce CBEs by blending these POP-rich fats with SOS-rich fats acquired by hexane fractionation of degummed shea butter. The DAG content in the as-prepared POP-rich fats was lower than that in the POP-rich fats produced by the previously reported two-step acetone fractionation [7]. The best process conditions, such as temperature, hexane quantity (for palm stearin only), and crystallization time, for the fractionation of palm stearin and degummed shea butter were established, and the optimal blending ratio of the POP- and SOS-rich fats achieved under the optimized conditions was determined by evaluating the melting and crystallization properties of these fats. Finally, the CBEs were blended with cocoa butter at several different weight ratios, and the melting and crystallization properties and solid-fat content (SFC) of the blends were examined.

## 2. Results and Discussion

### 2.1. Preparation of Low-DAG POP-Rich Fats from Palm Stearin

#### 2.1.1. Best Conditions for the First Fractionation Step

Herein, two-step hexane fractionation of palm stearin was performed to prepare POP-rich fats with lower levels of DAGs than those in the POP-rich fats prepared by previously reported two-step acetone fractionation of palm stearin [7]. The first step in fractionation was to obtain a liquid fraction without tripalmitin (PPP). Palm stearin containing 39.2 *w/w*% total SMUTs (31.4 *w/w*% POP, 5.1 *w/w*% POS, and 2.7 *w/w*% SOS), 15.2 *w/w*% PPP, and 3.0 *w/w*% DAGs was used as the starting material for fractionation. Note that the contents of SMUTs in the lipid samples investigated in this study included the contents of their positional isomers, such as PPO (1,2-dipalmitoyl-3-oleoyl-glycerol) for POP, PSO (1-palmitoyl-2-stearoyl-3-oleoyl-glycerol) and SPO (1-stearoyl-2-palmitoyl-3-oleoyl-glycerol) for POS, and SSO (1,2-distearoyl-3-oleoyl-glycerol) for SOS, as isomeric TAGs could not be separated by the high-performance liquid chromatography (HPLC)–evaporative light scattering detector (ELSD) system employed herein. The effects of temperature, volume-to-weight ratio of hexane-to-palm stearin, and crystallization time on the yields of the fractions and the contents of DAGs, POP, and PPP in the fractions were investigated, and the optimal conditions were determined.

**Figure 1:**
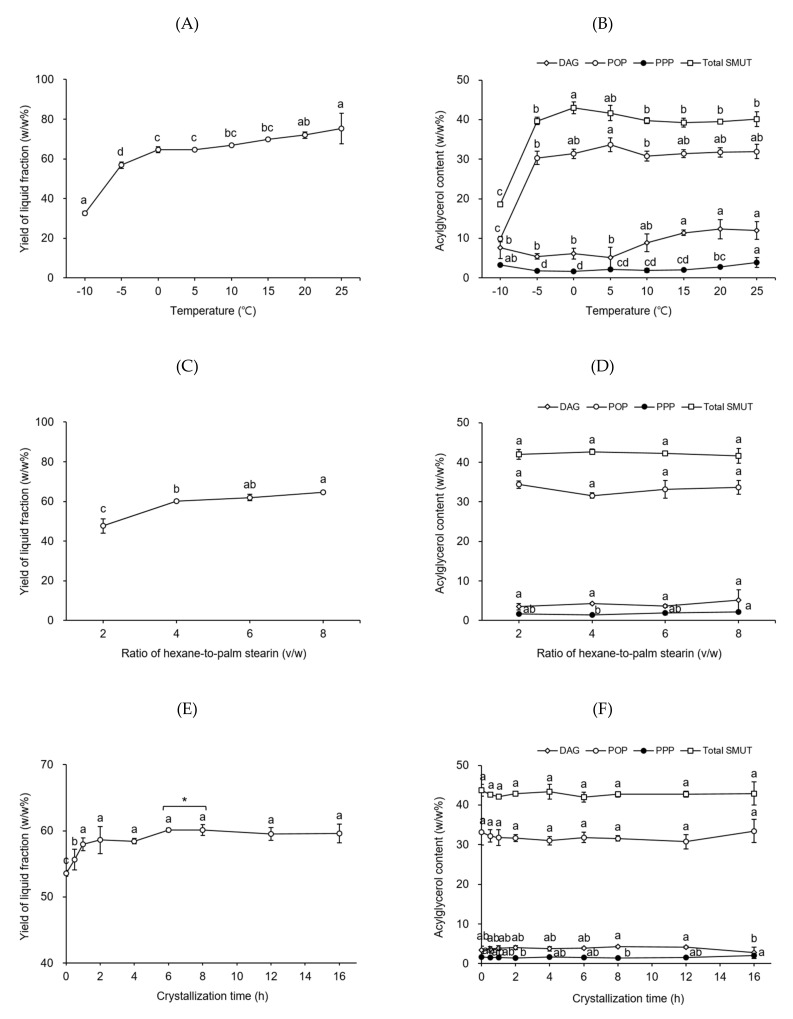
Yield of the liquid fraction (**A**,**C**,**E**) and the contents of diacylglycerols (DAGs), tripalmitin (PPP), 1,3-dipalmitoyl-2-oleoyl glycerol (POP), and total symmetric monounsaturated triacylglycerols (SMUTs) in the liquid fraction (**B**,**D**,**F**) obtained from palm stearin during the first hexane fractionation step as a function of temperature, volume-to-weight ratio of hexane-to-palm stearin, and crystallization time, respectively. Fractionation was performed, using 5 g palm stearin in a flat-bottom glass vessel (8 cm × 3.5 cm i.d.). Different letters (a–e) indicate significant differences between samples (*p* < 0.05). Asterisk (*) denotes significant differences (determined by a two-tailed Student’s *t*-test) between the two samples (*p* < 0.05).

Temperature is the most important factor for solvent fractionation of edible fats because the separation of higher-melting and lower-melting TAGs occurs based on the difference in their melting degrees and solubility at a specific temperature [13]. To determine the best temperature for the first fractionation step, the process was conducted by using a volume-to-weight ratio of hexane-to-palm stearin of 8 at desired temperatures (from −10 to 25 °C, at 5 °C intervals) for a crystallization time of 8 h. The yield of the liquid fraction acquired from palm stearin as a function of temperature is shown in Figure 1A. The yield of the liquid fraction gradually increased from 32.6 to 64.6 *w/w*% when the temperature was increased from −10 to 0 °C. In the temperature range 0–15 °C, no considerable difference was noticed in the yields of the liquid fraction (64.6–69.7 *w/w*%). Figure 1B shows the contents of DAGs, PPP, POP, and total SMUTs in the liquid fraction as a function of temperature. The PPP contents (1.7–2.1 *w/w*%) of the liquid fractions obtained at temperatures ranging from −5 to 15 °C were significantly lower than those of the liquid fractions (2.8–3.9 *w/w*%) produced at temperatures outside the range from −5 to 15 °C. The DAG contents (5.2–7.6 *w/w*%) of the liquid fractions acquired at temperatures in the range from −10 to 5 °C did not show a substantial difference; however, they were considerably lower than those of the liquid fractions (8.9–12.3 *w/w*%) obtained at 10–25 °C. These results suggest that the solubility of DAG in hexane significantly increases at temperatures above ~10 °C and fractionation should be performed at temperatures below ~10 °C to attain a liquid fraction with a lower content of DAGs. The maximum POP content (33.6 *w/w*%) was achieved at 5 °C. Thus, the best temperature for the first fractionation step was 5 °C. A possible approach to fabricate cost-effective CBE products by using targeted POP-rich fats obtained from palm stearin is to reduce the amount of hexane employed in the fractionation of palm stearin. To determine the most suitable volume-to-weight ratio of hexane-to-palm stearin for the first fractionation step, the process was performed, using different volume-to-weight ratios of hexane-to-palm stearin (2–8, at two intervals) at 5 °C for a crystallization time of 8 h. The yield of the liquid fraction obtained from palm stearin as a function of the volume-to-weight ratio of hexane-to-palm stearin is shown in Figure 1C. Although the yield of the liquid fraction attained at a ratio of 4 (60.1 *w/w*%) was not significantly different from that of the liquid fraction obtained at a ratio of 6 (61.9 *w/w*%), it was substantially lower than that of the liquid fraction achieved at a ratio of 8 (64.7 *w/w*%). The yield of the liquid fraction acquired at a ratio of 2 (47.7 *w/w*%) was considerably lower than those of the liquid fractions obtained at the ratios of 4–8. The contents of DAGs, PPP, POP, and total SMUTs in the liquid fraction as a function of the volume-to-weight ratio of hexane-to-palm stearin are shown in Figure 1D. No significant difference was found in the PPP contents (1.4–1.9 *w/w*%) of the liquid fractions attained at the ratios of 2–6. However, the PPP content of the liquid fraction achieved at a ratio of 4 (1.4 *w/w*%) was substantially lower than that of the liquid fraction acquired at a ratio of 8 (2.1 *w/w*%). There was no significant difference between the contents of DAGs (3.5–5.2 *w/w*%), POP (31.6–34.4 *w/w*%), and total SMUTs (41.6–42.7 *w/w*%) of all liquid fractions obtained at any specific ratio. Therefore, the most appropriate volume-to-weight ratio of hexane-to-palm stearin for the first fractionation step was 4.

Reducing the crystallization time for fractionation would help reduce the energy cost of the process, thereby resulting in the preparation of economic CBE products. To determine the best crystallization time for the first fractionation step, crystallization was conducted for 0.5–16 h at 5 °C and the volume-to-weight ratio of hexane-to-palm stearin of 4. Figure 1E depicts the yields of the liquid fractions attained from palm stearin as a function of crystallization time. A substantial increase in the yield of the liquid fractions was observed during the first 1 h of crystallization, whereas no significant difference was noticed in the yields (57.9–60.1 *w/w*%) of the liquid fractions during 1–16 h of crystallization. However, based on the two-tailed Student’s *t*-test results, the yield of the liquid fraction reached a constant value after 6 h of crystallization, and the yield of the liquid fraction acquired at 6 h (60.1 *w/w*%) of crystallization was considerably higher than that of the liquid fraction achieved at 4 h (58.4 *w/w*%) of crystallization. No substantial difference was noticed between the DAG (3.4–4.3 *w/w*%), PPP (1.4–1.6 *w/w*%), POP (30.8–33.2 *w/w*%), and total SMUT (42.0–43.8 *w/w*%) contents of all liquid fractions attained at different crystallization times, except for the fraction obtained at 16 h of crystallization (Figure 1F). Therefore, the crystallization time of 6 h was the best for the first fractionation step. Finally, PS-LF was acquired under the best conditions (i.e., temperature, 5 °C; volume-to-weight ratio of hexane-to-palm stearin, 4; and crystallization time, 6 h) established for the first fractionation step and used as the starting material for the second fractionation step.

#### 2.1.2. Best Conditions for the Second Fractionation Step

The objective of the second fractionation step was to achieve a solid fraction containing further increased level of POP compared to that in PS-LF. Best temperature and crystallization time for the second fractionation step were determined by investigating the effects of these parameters on the yields of the solid fractions and the contents of DAGs and POP in the fractions. In the second fractionation step, the quantity of hexane used was not a variable, but a constant, because PS-LF dissolved in hexane was used as the starting material for large-scale fractionation after establishing the best conditions for small-scale fractionation.

**Figure 2:**
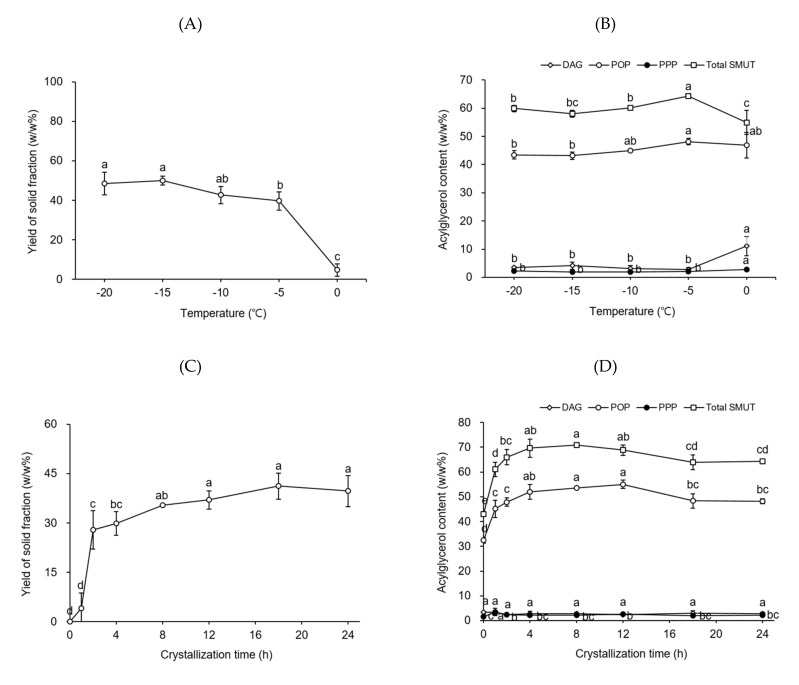
Yield of the solid fraction (**A**,**C**) and the contents of diacylglycerols (DAGs), tripalmitin (PPP), 1,3-dipalmitoyl-2-oleoyl glycerol (POP), and total symmetric monounsaturated triacylglycerols (SMUTs) in the solid fraction (**B**,**D**) obtained from PS-LF (a liquid fraction prepared by the first fractionation of palm stearin under the best conditions established in this study) during the second hexane fractionation step as a function of temperature and crystallization time, respectively. Fractionation was conducted by using 5 g PS-LF in a flat-bottom glass vessel (8 cm × 3.5 cm i.d.). Different letters (a–e) indicate significant differences between samples (*p* < 0.05).

To determine the best temperature for the second fractionation step, the process was conducted by using a volume-to-weight ratio of hexane-to-PS-LF of 4 at desired temperatures (from −20 to 0 °C, at 5 °C intervals) for a crystallization time of 24 h. Figure 2A shows the yield of the solid fraction acquired from PS-LF as a function of temperature. The yield of the solid fraction gradually decreased from 50.0 to 39.7 *w/w*% when the temperature was increased from −20 to −5 °C. The yield of the solid fraction obtained at 0 °C (4.8 *w/w*%) significantly decreased compared to that of the solid fraction attained at −5 °C (39.7 *w/w*%). The contents of DAGs, PPP, POP, and total SMUTs in the solid fraction as a function of temperature are shown in Figure 2B. The content of POP (48.2 *w/w*%) in the solid fraction achieved at −5 °C was maximum; however, the difference between the yields of the solid fractions produced at −10 °C (44.9 *w/w*%) and 5 °C (46.8 *w/w*%) was not statistically significant. The contents of DAGs (2.8–4.1 *w/w*%) in the solid fractions obtained at temperatures ranging from −20 to −5 °C were not considerably different; nevertheless, the DAG content was significantly and remarkably high in the solid fraction attained at 0 °C (11.1 *w/w*%) owing to reduction in the content of higher-melting SMUTs (such as POS) than that of POP. The POS content was in the range 12.3–13.6 *w/w*% in the solid fraction obtained at temperatures ranging from −20 to −5 °C, whereas it decreased to 5.6 *w/w*% in the solid fraction achieved at 0 °C. Thus, −5 °C was determined to be the best temperature for the second fractionation step.

To discover the best crystallization time for the second fractionation step, crystallization was performed for 1–24 h, using a volume-to-weight ratio of hexane-to-PS-LF of 4 at −5 °C. Figure 2C shows the yield of the solid fraction obtained from PS-LF as a function of crystallization time. The yields of the solid fractions substantially increased during the first 12 h of crystallization, whereas no significant difference was observed in the yields (37.0–41.2 *w/w*%) of the solid fractions acquired at a crystallization time of 12–24 h. Figure 2D shows the contents of DAGs, PPP, POP, and total SMUTs in the solid fractions as a function of crystallization time. The maximum POP content (55.0 *w/w*%) was achieved at 12 h of crystallization time. The DAG contents (2.4–3.4 *w/w*%) of the solid fractions did not considerably vary at all the crystallization times investigated herein. Thus, the best crystallization time for the second fractionation step was 12 h. Finally, PS-LF-SF was attained under the best conditions (i.e., a temperature of −5 °C and a crystallization time of 12 h) established for the second fractionation step.

### 2.2. Comparison between the POP-Rich Fats Prepared by Hexane Fractionation and Acetone Fractionation of Palm Stearin

**Figure 3:**
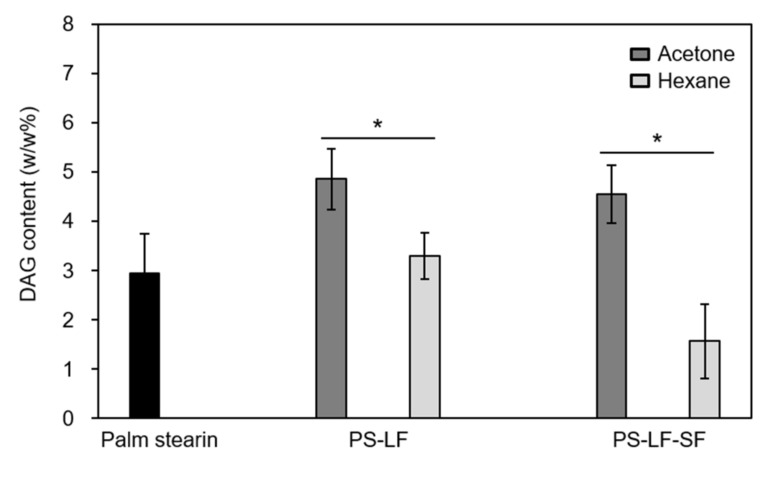
Comparison between the contents of diacylglycerols (DAGs) in fractions obtained by the two-step hexane fractionation of palm stearin and in the counterpart fractions prepared by the two-step acetone fractionation of palm stearin. Hexane fractionation was performed in a flat-bottom glass vessel (11 cm × 10 cm i.d.) under the best conditions established for the small-scale process, using another flat-bottom glass vessel (8 cm × 3.5 cm i.d.). Acetone fractionation was conducted in a flat-bottom glass vessel (11 cm × 10 cm i.d.) under the best conditions reported by Kang et al. [7]. Asterisk (*) indicates significant differences between the two samples (*p* < 0.05).

To investigate whether the two-step hexane fractionation method established in this study was effective in reducing the level of DAGs in PS-LF-SF, large-scale fractionation using ten times (i.e., 50 g) the quantity of palm stearin (DAG content = 3.0 *w/w*%) used in the small-scale fractionation was performed to prepare fractions including PS-LF-SF; moreover, the DAG contents of the fractions were compared with those of the counterpart fractions acquired by the previously reported two-step acetone fractionation of the same quantity of palm stearin [7] (Figure 3). Via acetone fractionation, PS-LF was achieved from palm stearin under the following best conditions established in the corresponding study: temperature = 17 °C, volume-to-weight ratio of acetone-to-palm stearin = 8, and crystallization time = 8 h. Subsequently, PS-LF-SF was obtained from PS-LF under the following best conditions established in the corresponding studies: a temperature of 4 °C and a crystallization time of 24 h. PS-LF attained via the first hexane fractionation step contained a significantly lower level (3.3 *w/w*%) of DAGs than that in the counterpart fraction (DAG content = 4.9 *w/w*%) produced by the first acetone fractionation step. These phenomena were ascribed to the higher concentration of DAGs in PS-SF produced along with PS-LF during the first hexane fractionation step (DAG content = 3.1 *w/w*%) than that in PS-SF obtained by the first acetone fractionation step (DAG content <0.05 *w/w*%); this indicated that DAGs were rarely removed from PS-LF during the first acetone fractionation step. These results are in accordance with those reported in previous studies [9,10], stating that hexane tends to concentrate more polar lipids, such as DAGs, in the solid phase rather than in the liquid phase compared to acetone because DAGs are less soluble in hexane than in acetone. Then, PS-LF-SF with a considerably lower level (1.6 *w/w*%) of DAGs was acquired from PS-LF by the second hexane fractionation step, whose DAG level was lower than that in the counterpart fraction (DAG content = 4.6 *w/w*%) obtained by the second acetone fractionation step. However, PS-LF-LF achieved along with PS-LF-SF by the second hexane fractionation step also contained significantly lower level of DAGs (DAG content = 7.0 *w/w*%) than that in the counterpart fraction (DAG content = 14.2 *w/w*%) produced by the second acetone fractionation step. These results suggested that unlike the case of the first fractionation step, no distinct tendency to concentrate DAG toward the solid fraction was observed during the second hexane fractionation step compared with the case of the second acetone fractionation step. These phenomena were attributed to the crystallization of a considerable portion of DAGs at 4 °C during the second acetone fractionation step compared to that in the case of the first acetone fractionation step at 17 °C. Therefore, these findings imply that the two-step hexane fractionation method proposed in this study is a more effective approach for the preparation of POP-rich fats containing a lower level of DAGs from palm stearin than the previously reported two-step acetone fractionation procedure.

### 2.3. Preparation of SOS-Rich Fats from Shea Butter

Hexane fractionation of the shea butter acquired after degumming was performed to prepare SOS-rich fats. Herein, the term “degumming” refers to the removal of gummy unsaponifiable substances (e.g., tocopherols) rather than the removal of phosphatides, which are typical gums present in common edible fats and oils. Shea butter contains high levels of unsaponifiable substances (5–10 *w/w*%) [11,14]. For the application of shea butter as an ingredient in chocolate manufacture, removal of the gummy unsaponifiable substances from the fat is necessary because they disturb the proper tempering of cocoa butter, and consequently, the chocolate products do not acquire desirable sensory attributes [11]. Furthermore, in this study, it was observed that when raw shea butter without degumming was used as the starting material for fractionation, it was not crystallized in hexane. In addition, acetone cannot be applied as a solvent for fractionation because both raw and degummed shea butter are insoluble in acetone. Degummed shea butter comprising 37.7 *w/w*% total SMUTs (2.9 *w/w*% POP, 4.8 *w/w*% POS, and 29.9 *w/w*% SOS) and 1.8 *w/w*% DAGs was used as the starting material for fractionation. Best temperature and crystallization time for the fractionation of degummed shea butter were determined by examining the effects of temperature and crystallization time on the yields of the fractions and the contents of DAG and SOS in the fractions. Unlike the case of the fractionation of palm stearin for the preparation of PS-LF-SF, the best volume-to-weight ratio of hexane-to-degummed shea butter was not determined herein. Instead, the volume-to-weight ratio of hexane-to-degummed shea butter was fixed at 8, which was the same as the volume-to-weight ratio of hexane-to-shea butter used for degumming. Therefore, SOS-rich fats were directly acquired from the degummed shea butter existing in the liquid phase after degumming; nevertheless, herein, hexane was removed after degumming to establish the best temperature and crystallization time conditions for the fractionation of degummed shea butter.

**Figure 4:**
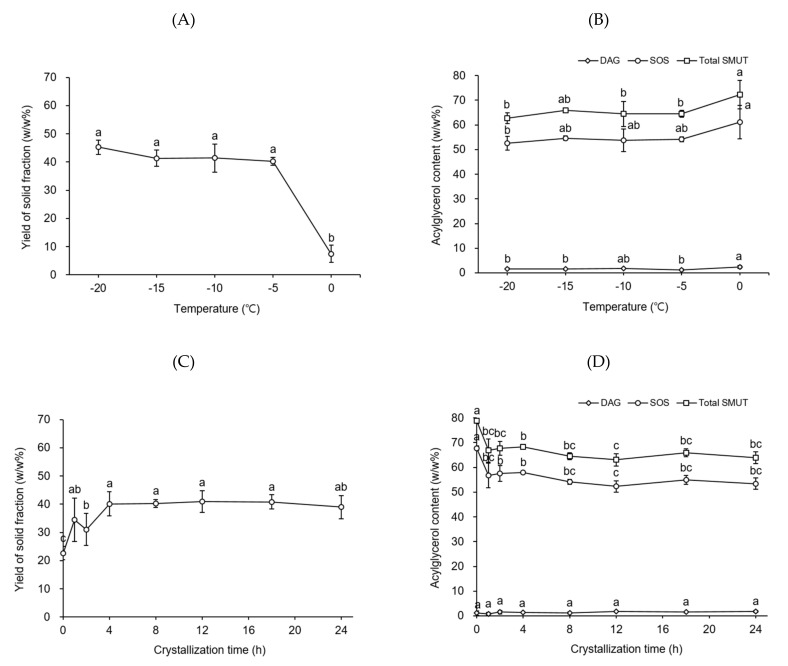
Yield of the solid fraction (**A**,**C**) and the contents of diacylglycerols (DAGs), 1,3-distearoyl-2-oleoyl glycerol, and total symmetric monounsaturated triacylglycerols (SMUTs) in the solid fraction (**B**,**D**) obtained by the hexane fractionation of degummed shea butter as a function of temperature and crystallization time, respectively. Fractionation was performed by using 5 g of degummed shea butter in a flat-bottom glass vessel (8 cm × 3.5 cm i.d.). Different letters (a–e) indicate significant differences between samples (*p* < 0.05).

To determine the best temperature for fractionation, the process was conducted with a volume-to-weight ratio of hexane-to-degummed shea butter of 8 at desired temperatures (from −20 to 0 °C, at 5 °C intervals) for a crystallization time of 24 h. Figure 4A shows the yield of the solid fraction acquired from degummed shea butter as a function of temperature. No significant difference was found between the yields of the solid fractions (40.3–45.2 *w/w*%) acquired at temperatures ranging from −20 to −5 °C. However, the yield of the solid fraction obtained at 0 °C (7.5 *w/w*%) was substantially lower than that of the solid fraction attained at −5 °C (40.3 *w/w*%). Degummed shea butter did not crystallize at temperatures >0 °C. No considerable difference was observed between the SOS (52.6–54.2 *w/w*%) and DAG (1.2–1.8 *w/w*%) contents of the solid fractions achieved at temperatures ranging from −20 to −5 °C (Figure 4B). Therefore, −5 °C was considered the best temperature for fractionation.

To discover the best crystallization time for fractionation, crystallization was performed for 1–24 h, using a volume-to-weight ratio of hexane-to-degummed shea butter of 8 at −5 °C. The yields of the solid fractions significantly increased during the first 4 h of crystallization, whereas no substantial difference was noticed in the yields of the solid fractions obtained after 4 h of crystallization (39.0–40.9 *w/w*%) (Figure 4C). Among the solid fractions achieved at the crystallization times of 4–24 h, the solid fraction attained at 4 h had a significantly higher SOS content (57.9 *w/w*%) than those of the other fractions (52.3–54.9 *w/w*%); in contrast, no considerable difference was found in the DAG contents of all the fractions (1.3–1.8 *w/w*%) (Figure 4D). Thus, 4 h was determined to be an optimal crystallization time for fractionation. Finally, shea stearin was acquired under the abovementioned best conditions (i.e., a temperature of −5 °C and a crystallization time of 4 h) established for fractionation. Large-scale fractionation using ten times (i.e., 50 g) the quantity of degummed shea butter used in the small-scale fractionation was conducted to fabricate shea stearin for the following analysis.

### 2.4. Preparation of CBEs by Blending PS-LF-SF with Shea Stearin

**Figure 5:**
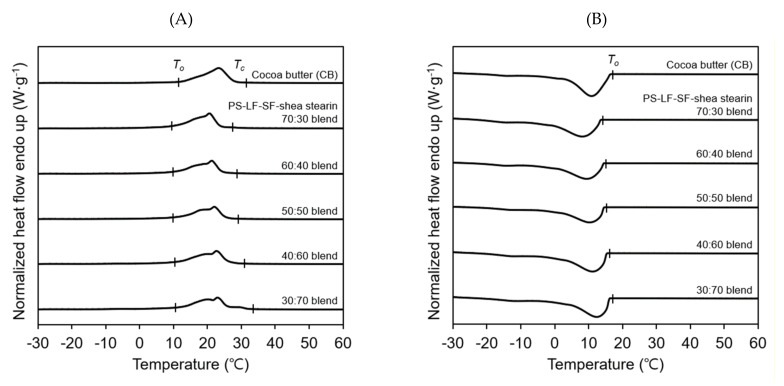
DSC melting (**A**) and crystallization (**B**) profiles of PS-LF-SF (POP-rich fats obtained by two-step hexane fractionation of palm stearin under the best conditions established in this study)–shea stearin (SOS-rich fats obtained by hexane fractionation of degummed shea butter under the best conditions optimized herein) blends with different PS-LF-SF:shea stearin weight ratios ranging from 70:30 to 30:70. *T*_o_ and *T*_c_ represent the onset and completion temperatures of melting or crystallization, respectively.

To establish the best blending ratio of PS-LF-SF and shea stearin for the preparation of CBEs, five different types of PS-LF-SF–shea stearin blends with PS-LF-SF:shea stearin weight ratios of 70:30, 60:40, 50:50, 40:60, and 30:70 were fabricated, and their differential scanning calorimetry (DSC) melting and crystallization profiles were compared with those of cocoa butter. The range of the blending ratios of PS-LF-SF and shea stearin was determined based on the results of preliminary examinations. The blends contained similar levels of total SMUTs (62.1–63.7 *w/w*%) and DAGs (1.2–1.6 *w/w*%) (data not shown here). The melting temperature ranges (i.e., from the onset to the completion temperatures of melting) of the blends containing larger quantities of shea stearin were lower (Figure 5A). The lower melting temperature range of the blends is in accordance with the lower quantity of high-melting TAGs, including SMUTs, in the blends than that in cocoa butter (77.9 *w/w*%). The melting temperature range of all the blends, including the 40:60 blend (10.5–30.8 °C), was significantly lower than that of cocoa butter (11.4–31.5 °C), except for the 30:70 blend (10.7–33.2 °C). Unlike the other four blends showing melting events consisting of two peaks, the 30:70 blend had an additional small peak at approximately 30 °C. This different melting profile of the 30:70 blend from those of the other blends caused the higher melting completion temperature of this blend than that of cocoa butter, which might result in incomplete melting of this blend at a temperature where the cocoa butter is completely melted. Figure 5B shows a comparison between the crystallization profiles of the various blends. In the crystallization profile, the crystallization temperature range was not determined because recognition of peak and baseline regions at around 0–5 °C at which a crystallization completion temperature is assumed to lie was extremely difficult. Thus, only the crystallization onset temperatures of the blends were compared. The crystallization onset temperatures of the blends containing larger quantities of shea stearin were higher; however, all the blends, such as the 40:60 blend (15.8 °C) and 30:70 blend (16.7 °C), demonstrated significantly lower crystallization onset temperatures than that of cocoa butter (17.0 °C). Overall, it was concluded that among the five types of PS-LF-SF–shea stearin blends, the 40:60 blend had the most comparable melting and crystallization profiles with those of cocoa butter.

### 2.5. Chemical Composition of CBEs

DAG contents and TAG compositions of the CBEs prepared by using the 40:60 PS-LF-S–shea stearin blend were compared with those of cocoa butter (Table 1). The DAG contents and TAG compositions of PS-LF-SF and shea stearin used for the synthesis of CBEs are also presented in the table. The TAG content shown in the table includes the contents of the positional isomers of TAGs, as described in Section 2.1.1. The DAG content of the CBEs (1.3 *w/w*%) was not significantly different from that of cocoa butter (1.1 *w/w*%). Kang et al. [7] prepared CBEs by blending POP-rich fats obtained by two-step acetone fractionation of palm stearin with shea stearin, and the resultant CBEs comprised a considerably higher DAG content (2.3 *w/w*%; analyzed by GC) than that of the cocoa butter (1.3 *w/w*%; analyzed by GC) used in their study. These results suggest that via two-step hexane fractionation of palm stearin, low-DAG POP-rich fats can be produced from palm stearin and subsequently utilized to fabricate CBEs containing a similar level of DAG to cocoa butter. The CBEs comprised 22.0 *w/w*% POP, 8.6 *w/w*% POS, and 33.1 *w/w*% SOS, with a total SMUT content of 63.7 *w/w*%. The total SMUT content of the CBEs was substantially lower than that of cocoa butter (75.9 *w/w*%). The presence of large amounts of TAGs, such as PPP and tristearin (SSS) with higher melting points than those of SMUTs causes incomplete melting of chocolate products [15]. Although PPP was removed via the first hexane fractionation of palm stearin, the PPP content in CBEs (2.9 *w/w*%) was significantly higher than that in cocoa butter (2.3 *w/w*). However, no considerable difference was found between the SSS contents of the CBEs (3.0 *w/w*%) and cocoa butter (2.5 *w/w*%). Because of the relatively lower content of SMUTs and higher content of SSS in CBEs than those in cocoa butter, it was expected that the CBEs prepared in this study would not satisfy the theoretical physical properties of CBEs, i.e., the ability to blend with cocoa butter in any proportion without altering the physical properties of cocoa butter.

Total FA composition and positional composition of FAs in CBEs and cocoa butter are provided in Table 2. The contents of two saturated FAs consisting of SMUTs, viz palmitic (16:0) and stearic acids (18:0), were substantially lower in CBEs (23.0 *w/w*% for 16:0 and 35.1 *w/w*% for 18:0) than those in cocoa butter (26.0 *w/w*% for 16:0 and 35.9 *w/w*% for 18:0), whereas the content of oleic acid (18:1*n*-9) in the CBEs (35.5 *w/w*%) was considerably higher than that in cocoa butter (32.8 *w/w*%). In both CBEs and cocoa butter, as expected, 16:0 and 18:0 were predominantly located at the *sn*-1,3 positions, whereas 18:1*n*-9 mainly existed at the *sn*-2 position. The tendency for this specific positional distribution of FAs was less pronounced in CBEs than that in cocoa butter. In other words, in CBEs, the contents of 16:0 (30.7 *w/w*%) and 18:0 (49.6 *w/w*%) at the *sn*-1,3 positions were ~3.9 and ~8.3 times higher those at the *sn*-2 position (7.8 *w/w*% for 16:0 and 6.0 *w/w*% for 18:0), respectively. In contrast, the contents of 16:0 (38.2 *w/w*%) and 18:0 (52.8 *w/w*%) at the *sn*-1,3 position of cocoa butter were ~22.5 and ~25.1 times higher than those at the *sn*-2 position (1.7 *w/w*% for 16:0 and 2.1 *w/w*% for 18:0), respectively. These results suggest the possibility of a more prevalent presence of SMUT positional isomers in CBEs than in cocoa butter.

### 2.6. Melting and Crystallization Properties of Cocoa Butter–CBE Blends

**Figure 6:**
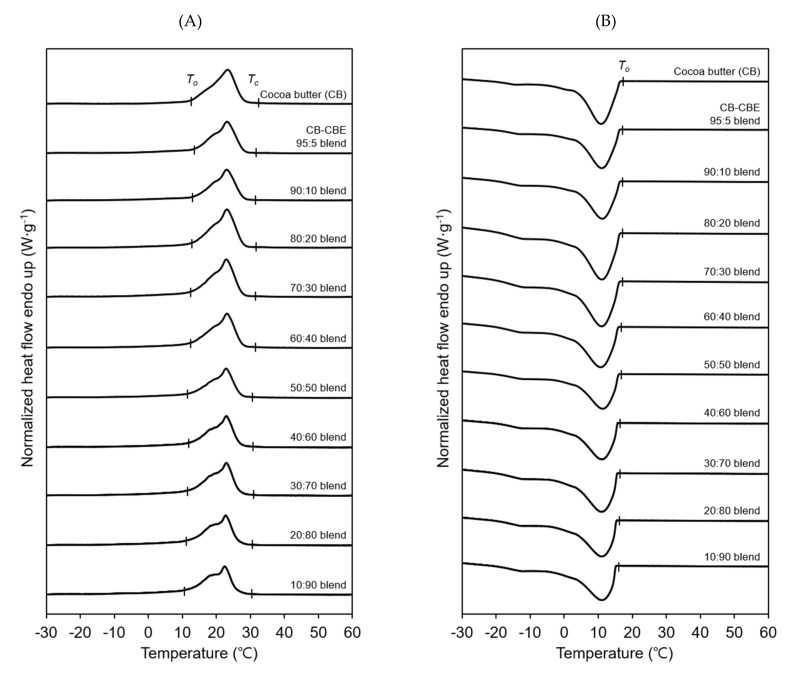
DSC melting (**A**) and crystallization (**B**) profiles of cocoa butter and cocoa butter–cocoa butter equivalent (CBE) blends with different cocoa butter:CBE weight ratios ranging from 95:5 to 10:90. *T*_o_ and *T*_c_ indicate the onset and completion temperatures of melting or crystallization, respectively.

Melting and crystallization properties of cocoa butter and cocoa butter–CBE blends with different cocoa butter:CBE weight ratios of 95:5, 90:10, 80:20, 70:30, 60:40, 50:50, 40:60, 30:70, 20:80, and 10:90 were compared, using DSC thermal profiles. The melting temperature ranges of the cocoa butter–CBE blends containing larger quantities of CBEs were significantly lower than that of cocoa butter (Figure 6A). Moreover, the cocoa butter–CBE blends comprising larger quantities of CBEs had considerably lower melting enthalpies (e.g., 60.4 J g^−1^ for the 95:5 blend and 54.2 J g^−1^ for the 10:90 blend), which were substantially lower than that of cocoa butter (68.0 J g^−1^) (data not shown here). Compared to the DSC melting profile of cocoa butter, the DSC melting profile of the cocoa butter–CBE blends had a shoulder peak at temperatures lower than those at which the main peak was found, and the intensity of this shoulder peak was higher for the blends containing larger quantities of CBEs. Figure 6B shows that the crystallization of the cocoa butter–CBE blends comprising more CBEs started at significantly lower temperatures, and the crystallization onset temperature of all the blends was considerably lower than that of cocoa butter. The lower melting temperature range, the more distinctive difference in the melting peak shapes, and the lower crystallization onset temperature of the cocoa butter–CBE blends containing higher quantities of CBEs imply that the addition amount of CBEs to cocoa butter should be optimized such that the CBEs blend with cocoa butter without altering the melting and crystallization properties of cocoa butter.

### 2.7. SFC of Cocoa Butter–CBE Blend

**Figure 7:**
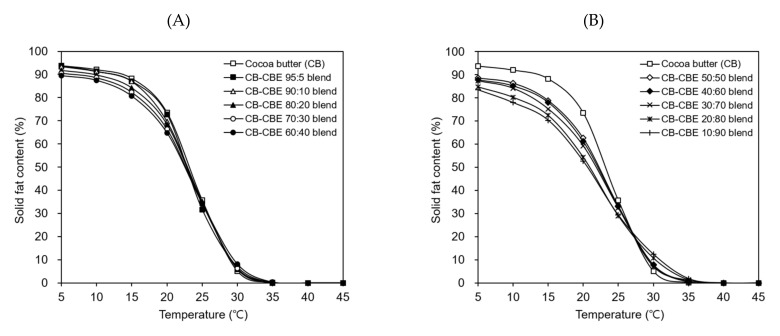
Solid-fat content of cocoa butter and cocoa butter–cocoa butter equivalent (CBE) blends with the cocoa butter:CBE weight ratios ranging from 95:5 to 60:40 (**A**) and from 50:50 to 10:90 (**B**).

SFC of cocoa butter at different temperatures is a crucial factor determining the physical and textural properties (such as hardness and melting behavior) of chocolate products [8]. The SFCs of cocoa butter and cocoa butter–CBE blends with the cocoa butter:CBE weight ratios ranging from 95:5 to 10:90 were compared as a function of temperature (5–45 °C). Although the SFCs of the cocoa butter–CBE blends were slightly lower than that of cocoa butter below 20 °C, the SFCs of the cocoa butter–CBE blends containing 5–40 *w/w*% CBEs showed similar changes to that of cocoa butter in the temperature range examined herein (Figure 7A). In other words, the SFCs of the cocoa butter–CBE blends were over 80% at 5–15 °C, thereby indicating the high melting resistance of the blends in this temperature range. Furthermore, the SFCs of the blends sharply decreased at 20–30 °C (suggesting rapid melting of the blends in this temperature range) and finally reached zero at 40 °C (implying complete melting of the blends at this temperature). In contrast, the cocoa butter–CBE blends containing 50–90 *w/w*% CBEs had different SFC profiles from that of cocoa butter; i.e., the SFCs of the blends were below 80% at 15 °C and relatively gently decreased at 20–30 °C (Figure 7B). These results suggested that the CBEs prepared in this study blended with cocoa butter at levels of up to 40 *w/w*% without distinctly altering the hardness and melting behavior of cocoa butter.

## 3. Materials and Methods

### 3.1. Materials

Spanish cocoa butter was purchased from Sunin Co. (Yongin, Korea). Palm stearin and shea butter were obtained from Ottogi Co. (Anyang, Korea) and Korea Similac (Pochun, Korea), respectively. HPLC-grade *n*-hexane (>99.5%), acetone (>99.3%), acetonitrile (>99.9%), and dichloromethane (>99.9%) were procured from J.T. Baker (Phillipsburg, NJ, USA). Silica gel–coated glass plates (20 cm × 20 cm, 0.25 mm thick) for thin-layer chromatography (TLC) were purchased from Merck KGaA (Darmstadt, Germany). Pancreatic lipase was acquired from Sigma Chemical Co. (St. Louis, MO, USA). Triolein (OOO; ≥99%), 1,2-dioleoyl-3-palmitoyl-*rac*-glycerol (POO; ≥99%), POP (≥99%), and PPP (≥99%), used as TAG standards, were also purchased from Sigma Chemical Co. All other reagents used herein were HPLC-grade.

### 3.2. Fractionation of Palm Stearin

Herein, two-step hexane fractionation of palm stearin was performed in a flat-bottom glass vessel (8 cm × 3.5 cm i.d.) equipped with a water jacket for temperature control to prepare POP-rich fats (sn−1,3 A). For the first fractionation step, palm stearin (5 g) with the desired quantity of hexane (10–40 mL) was added to the vessel, which was then heated to 45 °C, using a water circulator (RW-0252G, Jeio Tech, Seoul, Korea) to completely melt palm stearin in hexane. The resulting mixture was stirred by using a magnetic stirrer (300 rpm) at a desired temperature (ranging from −10 to 25 °C), for a desired time period (0–16 h). The fractionation products were filtered through a Buchner funnel, using Whatman filter paper no. 41 to separate the liquid and solid phases. The liquid phase was dried in a rotary vacuum evaporator at 60 °C and further dried under a N_2_ flush to obtain a liquid fraction. The liquid fraction achieved under the best conditions for the first fractionation step was designated as PS-LF. Optimal temperature, volume-to-weight ratio of hexane-to-palm stearin, and crystallization time were established by considering the yields of the fractions and the contents of DAGs, POP, and PPP in the fractions. Second fractionation step, i.e., fractionation of PS-LF, was conducted in the same manner as the first fractionation step. PS-LF (5 g) was crystallized in 20 mL hexane under stirring at 300 rpm at a desired temperature (ranging from −20 to 0 °C) for a desired time period (0–24 h). The solid fraction acquired under the best conditions for the second fractionation step was designated as PS-LF-SF. Optimal temperature and crystallization time were determined by considering the yields of the fractions and the contents of DAG and POP in the fractions. Large-scale fractionation of palm stearin (50 g) was performed in a flat-bottom glass vessel (11 cm × 10 cm i.d.) under the best conditions established for the abovementioned small-scale process to fabricate CBEs by blending POP-rich fats with the SOS-rich fats obtained by the fractionation of shea butter. For the second step of large-scale fractionation, PS-LF dissolved in hexane was used as the starting material.

### 3.3. Fractionation of Shea Butter

**Figure 8:**
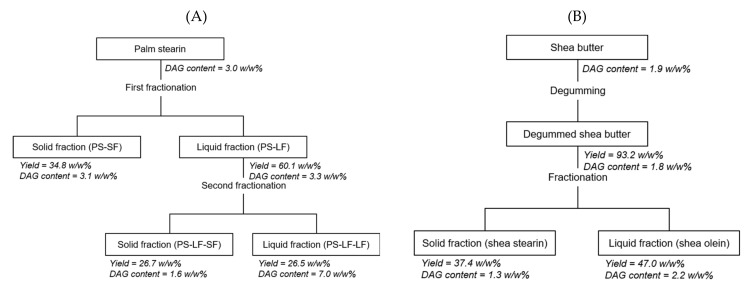
Preparation of 1,3-dipalmitoyl-2-oleoyl glycerol-rich fats by two-step hexane fractionation of palm stearin (**A**) and 1,3-distearoyl-2-oleoyl glycerol-rich fats by degumming of shea butter followed by hexane fractionation of the degummed shea butter (**B**). Fractionation was performed in a flat-bottom glass vessel (11 cm × 10 cm i.d.), using 50 g palm stearin or degummed shea butter under the best conditions established for the small-scale process, using another flat-bottom glass vessel (8 cm × 3.5 cm i.d.). The yield of each fraction was expressed as a percentage of the initial palm stearin weight or shea butter weight. The diacylglycerol (DAG) content of each fraction was expressed as a percentage of the weight of the fraction.

Fractionation of the shea butter achieved after degumming was conducted to acquire shea stearin, which is rich in SOS (Figure 8B). Degumming of shea butter was performed according to a previously reported method [11] with a slight modification. Shea butter (5 g) and hexane (40 mL) were placed in a flat-bottom glass vessel (8 cm × 3.5 cm i.d.). After complete melting of shea butter in hexane at 45 °C, the mixture was stirred by using a magnetic stirrer (300 rpm) at 5 °C for 2 h. The resultant products were filtered through a Buchner funnel with Whatman filter paper no. 41 (Pall Corporation, Ann Arbor, MI, USA) to obtain the filtrate. Then, the filtrate was dried in a rotary vacuum evaporator at 60 °C and further dried under N_2_ flush to acquire degummed shea butter. For fractionation, degummed shea butter (5 g) was crystallized in hexane (40 mL) under stirring at 300 rpm at a desired temperature (ranging from −20 to 0 °C) for a desired time period (0–24 h). The solid fraction attained under the best conditions for fractionation was denoted as shea stearin. The best temperature and crystallization time were determined by considering the yields of the fractions and the content of SOS in the fractions. Large-scale fractionation of degummed shea butter (50 g) was conducted in a flat-bottom glass vessel (11 cm × 10 cm i.d.) under the optimal conditions established for the abovementioned small-scale fractionation to obtain shea stearin in sufficient quantity for blending with PS-LF-SF for the preparation of CBEs.

### 3.4. Preparation of CBEs and Cocoa Butter–CBE Blends

PS-LF-SF and shea stearin were blended in PS-LF-SF:shea stearin weight ratios of 70:30, 60:40, 50:50, 40:60, and 30:70. The best blending ratio (40:60) of PS-LF-SF and shea stearin was determined by comparing the DSC melting and crystallization profiles of the blends with those of cocoa butter. CBEs were prepared by blending PS-LF-SF with shea stearin in the best blending ratio, and were stored at −20 °C prior to analysis. Cocoa butter was blended with the CBEs in cocoa butter:CBE weight ratios of 95: 5, 90:10, 80:20, 70:30, 60:40, 50:50, 40:60, 30:70, 20:80, and 10:90. The resulting cocoa butter–CBE blend samples were stored at −20 °C prior to analysis.

### 3.5. FA Composition

Total FAs composition of the lipid samples was analyzed according to a previously reported method [7]. Lipid samples (20 mg) were saponified with 3 mL of 0.5 N methanolic sodium hydroxide solution at 85 °C for 10 min, cooled to room temperature, and then methylated with 3 mL of 14% methanolic boron trifluoride solution at 85 °C for 10 min. After cooling the resulting samples to room temperature, 3 mL isooctane and 5 mL saturated NaCl solution were added to it, and then, the mixture was vortexed. The upper isooctane layer containing FA methyl esters (FAME) was collected and passed through an anhydrous sodium sulfate column. FAMEs were examined by gas chromatography (GC), using an Agilent Technologies 7890A gas chromatograph (Palo Alto, CA, USA) equipped with a flame ionization detector and a fused silica column (SP-2560, 100 m × 0.25 mm i.d. × 0.2 μm film thickness, Supelco, Bellefonte, PA, USA). FAME samples (1 μL) were injected into the GC system in split mode with a split ratio of 200:1. The carrier gas was He, and the flow rate was 1.0 mL min^−1^. Injector and detector temperatures were maintained at 225 and 285 °C, respectively. Oven temperature was initially set at 100 °C for 4 min, increased to 240 °C at a rate of 3 °C min^−1^, and finally maintained at 240 °C for 17 min. FAMEs were identified by comparing their retention times with those of the standards, and their relative contents were calculated as *w/w*%. Compositions of the FAs at the *sn*-2 and *sn*-1,3 positions of TAG in lipid samples were determined according to a previously reported method [16]. Lipid samples (40 mg) were mixed with 2 mL of 1 M Tris–HCl buffer (pH: 7.8), 0.5 mL of 0.05 *w/v*% sodium cholate solution, and 0.2 mL of 2.2 *w/v*% calcium chloride solution. After thorough vortexing to emulsify the samples, 20 mg pancreatic lipase was added to the resulting samples. The mixtures were incubated in a water bath at 40 °C for 2 min and vigorously vortexed for 30 s, followed by the addition of 5 mL of 6 M hydrochloric acid. Anhydrous diethyl ether (15 mL × 2) was added to the abovementioned mixtures followed by vortexing. The upper diethyl ether layer containing pancreatic lipase hydrolysates was collected and passed through an anhydrous sodium sulfate column. Then, the hydrolysates were placed on a silica gel G TLC plate (Merck, Darmstadt, Germany) and developed with hexane/diethyl ether/acetic acid (50:50:1, *v*/*v*/*v*). The plate was dried in air and sprayed with 0.2 *w/v*% 2,7-dichlorofluorescein in methanol. The band corresponding to *sn*-2 monoacylglycerols was scraped from the plates, methylated, and analyzed by GC as previously reported to determine the composition of the FAs at the *sn*-2 position. The composition of FAs at the *sn*-1,3 position was evaluated by using the following Equation (1):

(1) $$sn-1,3\;\left(\frac{w}{w}\%\right)=\frac{\left[3\times \;\mathrm{total}\;\left(\frac{w}{w}\%\right)-sn-2\;\left(\frac{w}{w}\%\right)\right]}{2}$$

### 3.6. TAG Composition

**Figure 9:**
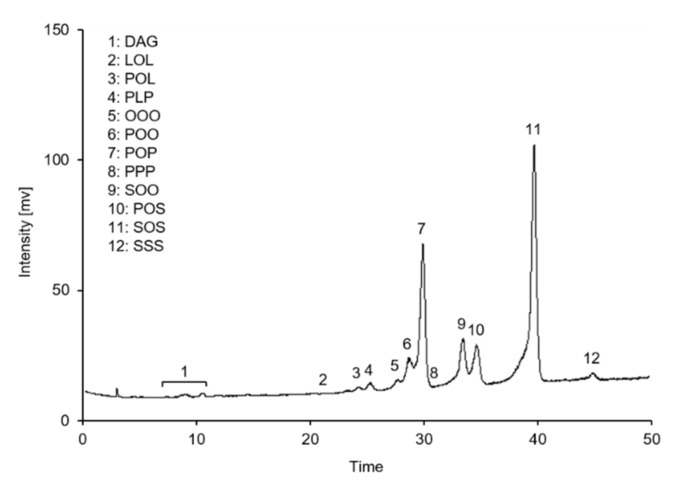
A typical high-performance liquid chromatography/evaporative light-scattering detector chromatogram of triacylglycerols contained in cocoa butter equivalents prepared in the present study.

Compositions of TAGs in the lipid samples were examined by using an HPLC system coupled with an ELSD, according to a previously reported method [17] with minor modifications. The HPLC system consisted of a pump (PU-2089) and an autosampler (AS-2057; JASCO Corp., Tokyo, Japan). The ELSD (Alltech^®^ 3300, Alltech Associates Inc., Deerfield, IL, USA) was operated at a drift tube temperature of 70 °C, a gain setting of 4, and a N_2_ gas flow rate of 2.5 L min^−1^. Separation of TAGs was performed by using a Develosil C30 column (250 mm × 4.6 mm; particle size, 5.27 μm; Nomura Chemical Co., Seto, Japan) and a mobile phase consisting of (A) acetonitrile and (B) dichloromethane at a flow rate of 1 L min^−1^. Lipid samples (1 mg) were dissolved in 4 mL dichloromethane and filtered through a 0.45 μm hydrophobic PTFE membrane (Pall Corporation, Ann Arbor, MI, USA), and the resultant solution was injected into the column for analysis. Herein, twelve TAGs were detected in the samples and identified by comparing their retention times with those of the corresponding standards (i.e., OOO, POO, POP, and PPP) analyzed in this study or according to previously reported results [7,8,17]. Each TAG was quantified by using a standard calibration curve constructed employing a serial dilution of TAG standards, viz OOO for triunsaturated TAGs (e.g., OOO and LOL), POO for diunsaturated TAGs (such as POO, palmitoyl-oleoyl-linolein (POL), dipalmitoyl-linolein (PLP), and dioleoyl-stearin (SOO)), POP for monounsaturated TAGs (including POP, POS, and SOS), and PPP for trisaturated TAGs (e.g., PPP, dipalmitoyl-stearin (PSP), and SSS). Subsequently, relative content of each TAG was calculated as *w/w*% of the total contents of TAGs and DAGs found in the samples. Figure 9 shows a typical HPLC–ELSD chromatogram of TAGs found in the lipid samples.

### 3.7. DAG Content

DAGs present in the lipid samples were separated by HPLC–ELSD as abovementioned in Section 2.6, and the DAG content was determined by using a standard calibration curve generated by using a DAG standard. The DAG standard was fabricated by using PS-LF-SF by pancreatic-lipase-catalyzed hydrolysis, followed by TLC separation and recovery of the DAG band, using diethyl ether as an extraction solvent (as abovementioned in Section 2.5). The DAG content was measured as *w/w*% of the total contents of TAGs and DAGs found in the samples.

### 3.8. Melting and Crystallization Temperatures and Enthalpies

DSC melting and crystallization temperatures and enthalpies of the lipid samples were evaluated by using a DSC 4000 (Perkin–Elmer Co., Shelton, CT, USA) according to AOCS Official Method Cj 1–94 [18]. Normal standardization was conducted by using indium with a melting point of 156.6 °C and a melting enthalpy of 28.45 J g^−1^ as a reference standard. Liquid N_2_ was used as a coolant. The flow of the coolant through the circulating chamber attached to the DSC system was controlled by Intracooler 2P (Perkin–Elmer Co., Shelton, CT, USA). Lipid samples (6–8 mg) were tightly sealed in a 50 μL Al pan, and an empty sealed pan was employed as a reference. These lipid samples were rapidly heated from room temperature to 80 °C and maintained at this temperature for 10 min before being cooled to −60 °C, at a rate of 10 °C min^−1^, to achieve the crystallization profile. After maintaining the samples at −60 °C for 30 min, the resulting samples were heated to 80 °C, at a rate of 5 °C/min, to obtain the melting profile. The profiles were analyzed by the software provided with the DSC system (Pyris software, Perkin–Elmer Co., Shelton, CT, USA).

### 3.9. SFC

SFC of the lipid samples was determined according to the AOCS official method Cd 16b-93 [18]. Nuclear magnetic resonance (NMR) spectroscopy was performed by using Minispec MQ20 (Bruker BioSpin, Billerica, MA, USA). NMR tubes (10 mm in diameter) were filled with lipid samples to a height of 4 cm. Thereafter, these samples were tempered at 100 °C for 15 min, at 65 °C for 1 h, at 0 °C for 1 h, and finally at each chosen measuring temperature for 30 min. SFC was determined at 5 °C intervals, from 5 to 45 °C.

### 3.10. Statistical Analysis

Statistical analysis was conducted using IBM SPSS Statistics (version 25) software (SPSS Inc., Chicago, IL, USA). All data are presented as the mean ± standard deviation. The difference between lipid samples was assessed by using a two-tailed Student’s *t*-test or one-way analysis of variance, followed by Duncan’s multiple range test. Differences were considered significant if the *p*-value was less than 0.05.

## 4. Conclusions

In this study, CBEs were prepared by blending POP-rich fats obtained by two-step hexane fractionation of palm stearin and SOS-rich fats acquired by hexane fractionation of degummed shea butter. This study demonstrated that the two-step hexane fractionation method is a more effective approach for the preparation of POP-rich fats containing a lower level of DAGs from palm stearin than the previously reported two-step acetone fractionation procedure. We established the best conditions (including temperature, hexane quantity, and crystallization time) for the fractionation of palm stearin to achieve desired levels of chemical constituents (such as DAGs, PPP, and POP), affecting the physical and textural properties of chocolate, in the resultant POP-rich fats. Similarly, for the fractionation of degummed shea butter, we determined the best conditions (including temperature and crystallization time) to prepare SOS-rich fats containing desired levels of DAGs and SOS. Accordingly, the CBEs fabricated in this study also contained comparable levels of DAGs to that of cocoa butter. Based on the SFC results, we concluded that up to 40 *w/w*% CBEs could be added to cocoa butter to attain the cocoa butter–CBE blend without significantly altering the physical properties (such as hardness and melting behavior) of cocoa butter for chocolate manufacture. The limit in the addition quantity of CBEs to cocoa butter was owing to the relatively lower level of SMUTs and a relatively higher level of SSS in CBEs than those in cocoa butter. Finally, this study contains limitations. The contents of individual TAG species in the CBEs reported by this study included the contents of their positional isomers, because isomeric TAGs could not be separated by the HPLC–ELSD system used in this study. Furthermore, although the existence of some more TAG species, such as stearoyl-oleoyl-linolein (SOL), is theoretically possible in the CBEs, they were not detected by using the HPLC–ELSD system. Further investigation on the separation and identification of TAGs in the CBEs by using mass spectrometry–based methods would be required to obtain the accurate TAG composition of the CBEs.

## Figures and Tables

**Table 1:** Diacylglycerol (DAG) content and triacylglycerol composition (*w/w*%) of cocoa butter, PS-LF-SF, shea stearin, and cocoa butter equivalents (CBEs).

Acylglycerol	Cocoa Butter	PS-LF-SF ^(1)^	Shea Stearin ^(2)^	CBEs ^(3)^
DAGs	1.1 ± 0.3 ^(4)^	2.2 ± 0.5	1.3 ± 0.2	1.3 ± 0.3
PSP	2.4 ± 0.1	3.5 ± 0.2		3.0 ± 0.3
PPP	2.3 ± 0.1	3.4 ± 0.2		2.9 ± 0.3 *
POP	17.1 ± 0.1	48.5 ± 0.8	2.9 ± 0.1	22.0 ± 0.4 *
PLP	3.1 ± 0.2	6.3 ± 0.1	2.8 ± 0.2	3.9 ± 0.2 *
POS	34.3 ± 0.4	9.6 ± 0.4	7.6 ± 0.4	8.6 ± 0.1 *
POO	2.6 ± 0.6	8.2 ± 0.5	2.5 ± 0.1	4.8 ± 0.4 *
POL	2.1 ± 0.3	4.0 ± 0.2	2.0 ± 0.2	2.8 ± 0.1 *
SSS	2.5 ± 0.1		2.5 ± 0.1	3.0 ± 0.3
SOS	24.5 ± 0.1	4.4 ± 0.2	57.9 ± 0.5	33.1 ± 1.1 *
SOO	3.1 ± 0.5	3.2 ± 0.2	15.6 ± 0.9	8.7 ± 0.3 *
OOO	2.5 ± 0.4	3.4 ± 0.1	2.7 ± 0.1	3.1 ± 0.2 *
LOL	2.3 ± 0.2	3.3 ± 0.2	2.4 ± 0.3	2.8 ± 0.3
Total SMUTs	75.9 ± 0.4	62.6 ± 0.8	68.4 ± 0.4	63.7 ± 1.0 *

^(1)^ POP-rich fats obtained by two-step hexane fractionation of palm stearin under the best conditions established in this study. ^(2)^ SOS-rich fats achieved by hexane fractionation of degummed shea butter under the best conditions determined herein. ^(3)^ Prepared by blending PS-LF-SF with shea stearin in a PS-LF-SF:shea stearin weight ratio of 40:60. ^(4)^ Mean ± SD (*n* = 3). * Significantly different from cocoa butter (*p* < 0.05).

**Table 2:** Total and positional composition (*w/w*%) of cocoa butter, PS-LF-SF, shea stearin, and cocoa butter equivalents (CBEs).

Fatty Acid ^(1)^	Cocoa Butter			PS-LF-SF ^(2)^			Shea Stearin ^(3)^			CBEs ^(4)^		
	Total	*sn*-2	*sn*-1,3	Total	*sn*-2	*sn*-1,3	Total	*sn*-2	*sn*-1,3	Total	*sn*-2	*sn*-1,3
12.0				0.3 ± 0.0 ^(5)^	0.4 ± 0.1	0.2 ± 0.0				0.1 ± 0.0	0.3 ± 0.0 *	
14:0	0.1 ± 0.0		0.1 ± 0.0	0.9 ± 0.0	0.4 ± 0.1	1.1 ± 0.0	0.1 ± 0.0		0.1 ± 0.0	0.4 ± 0.0	0.3 ± 0.0 *	0.4 ± 0.0 *
16:0 (P)	26.0 ± 0.1	1.7 ± 0.0	38.2 ± 0.0	52.7 ± 0.0	15.2 ± 0.4	71.5 ± 0.2	26.0 ± 0.1	1.7 ± 0.5	38.2 ± 0.3	23.0 ± 0.0 *	7.8 ± 0.5 *	30.7 ± 0.2 *
16:1	0.2 ± 0.0	0.4 ± 0.0	0.2 ± 0.0	0.1 ± 0.0	0.2 ± 0.1	0.1 ± 0.0	0.2 ± 0.0	0.4 ± 0.0	0.2 ± 0.0	0.1 ± 0.0	0.2 ± 0.0	
18:0 (S)	35.9 ± 0.2	2.1 ± 0.0	52.8 ± 0.0	5.1 ± 0.0	1.2 ± 0.1	7.1 ± 0.1	35.9 ± 1.5	2.1 ± 2.4	52.8 ± 1.3	35.1 ± 0.0 *	6.0 ± 2.4	49.6 ± 1.2 *
18:1*n*-9 (O)	32.8 ± 0.0	86.2 ± 0.1	6.1 ± 0.0	34.5 ± 0.0	71.3 ± 0.3	16.1 ± 0.1	32.8 ± 1.1	86.2 ± 2.8	6.1 ± 1.4	35.5 ± 0.0 *	75.4 ± 2.5 *	15.6 ± 1.3 *
18:1*n*-7	0.4 ± 0.0	0.6 ± 0.0	0.3 ± 0.0	0.6 ± 0.0	0.7 ± 0.0	0.5 ± 0.0	0.4 ± 0.0	0.6 ± 0.1	0.3 ± 0.1	0.4 ± 0.0	0.4 ± 0.0 *	0.3 ± 0.0
18:2*n*-6	3.0 ± 0.0	7.7 ± 0.1	0.6 ± 0.0	5.2 ± 0.0	10.2 ± 0.3	2.7 ± 0.1	3.0 ± 0.4	7.7 ± 1.0	0.6 ± 0.5	3.9 ± 0.0 *	8.6 ± 0.3 *	1.6 ± 0.1 *
20:0	1.1 ± 0.0	0.3 ± 0.0	1.5 ± 0.0	0.3 ± 0.0	0.1 ± 0.0	0.4 ± 0.0	1.1 ± 0.1	0.3 ± 0.2	1.5 ± 0.1	1.2 ± 0.0	0.3 ± 0.0	1.6 ± 0.0
20:1	0.1 ± 0.1	0.3 ± 0.1		0.1 ± 0.0	0.2 ± 0.0	0.1 ± 0.0	0.1 ± 0.0	0.3 ± 0.1		0.1 ± 0.0	0.2 ± 0.0	0.1 ± 0.0 *
18:3*n*-3	0.2 ± 0.0	0.4 ± 0.0	0.1 ± 0.0	0.1 ± 0.0	0.1 ± 0.0	0.1 ± 0.0	0.2 ± 0.0	0.4 ± 0.2	0.1 ± 0.0	0.1 ± 0.0	0.3 ± 0.1 *	
22:0	0.2 ± 0.0	0.3 ± 0.0	0.1 ± 0.0	0.1 ± 0.0		0.1 ± 0.0	0.2 ± 0.0	0.3 ± 0.1	0.1 ± 0.1	0.1 ± 0.0	0.2 ± 0.0	0.1 ± 0.0
P + S + O	94.7 ± 0.1	90.0 ± 0.1	97.1 ± 0.0	92.3 ± 0.0	87.7 ± 0.0	94.7 ± 0.0	94.7 ± 0.3	90.0 ± 1.0	97.1 ± 0.5	93.6 ± 0.0 *	89.2 ± 0.3 *	95.9 ± 0.2 *

^(1)^ P, palmitic; S, stearic; O, oleic acids. ^(2)^ POP-rich fats obtained by two-step hexane fractionation of palm stearin under the best conditions established in this study. ^(3)^ SOS-rich fats attained by hexane fractionation of degummed shea butter under the best conditions established herein. ^(4)^ Prepared by blending PS-LF-SF with shea stearin in a PS-LF-SF:shea stearin weight ratio of 40:60. ^(5)^ Mean ± SD (*n* = 3). * Significantly different from cocoa butter (*p* < 0.05)

## Data Availability

All relevant data are included in the article.

## Sample Availability

Samples of the compounds are not available from the authors.
